# The Potential of Cochlospermum tinctorium, Flueggea virosa, and Waltheria indica Traditional Plants From Burkina Faso in Treating Periodontitis: A Systematic Review

**DOI:** 10.7759/cureus.52471

**Published:** 2024-01-17

**Authors:** Abdoulaziz Diarra, Kevimy Agossa, Estelle Noëla Hoho Youl

**Affiliations:** 1 Periodontology, Training and Research Unit in Health Sciences (UFR/SDS) University of Ouaga I, Pr. Joseph Ki-Zerbo, Ouagadougou, BFA; 2 Periodontology, Lille University Hospital, Lille, FRA; 3 Pharmacology, Training and Research Unit in Health Sciences (UFR/SDS) University of Ouaga I, Pr. Joseph Ki-Zerbo, Ouagadougou, BFA

**Keywords:** treatment, periodontitis, waltheria indica, flueggea virosa, cochlospermum tinctorium

## Abstract

Periodontitis is a chronic, infectious, and inflammatory oral disease with a high prevalence in developing countries, where limited access to modern dental care curtails its treatment. This review is dedicated to examining three indigenous botanical species frequently recommended by traditional therapists for the treatment of periodontal disease, namely, *Cochlospermum tinctorium, Flueggea virosa, and Waltheria indica*, with the aim of elucidating their chemical constituents and pharmacological properties that may support their empirical use.

This review adheres to the Preferred Reporting Items for Systematic Reviews and Meta-Analyses (PRISMA) guidelines extension for scoping reviews. An electronic search was conducted in three databases (PubMed, Science Direct, and Google Scholar) up to July 2022. Out of 700 articles initially identified, only 11 were deemed eligible for inclusion; a substantial majority (80%) of these comprised in vitro studies. Among the trio of botanicals considered, *Waltheria indica* emerged as the most extensively investigated (65% of the studies). The administration of these plants was predominantly in the form of decoctions or macerations, with extraction methods employing alcoholic agents (ethanolic and methanolic), hydroalcoholic solutions, or aqueous solvents.

The selected plants exhibited notable richness in polyphenolic compounds, particularly flavonoids, and demonstrated anti-inflammatory effects, as indicated in 60% of the studies, along with antibacterial properties (against *Streptococcus aureus and Helicobacter pylori*). None of the studies reported antibacterial activity against periodontal pathogens.

The pharmacological properties of these plants may hold promise for the management of oral inflammatory and infectious conditions. Nevertheless, further comprehensive investigations are imperative to establish their safety and efficacy for periodontitis treatment before conclusive recommendations can be formulated.

## Introduction and background

Periodontitis is a chronic, non-communicable inflammatory disease arising from oral microbiota dysbiosis. It leads to irreversible local damage to the tissues supporting the tooth, potentially culminating in tooth loss if left untreated. Notably, periodontitis exerts a substantial impact on patients' quality of life and increases the risk of major systemic diseases, including diabetes, cardiovascular diseases, and adverse pregnancy outcomes [1]. Global disparities in the prevalence and severity of periodontitis are associated with socioeconomic factors [2]. Periodontitis has been found to be more prevalent and severe in low-income countries, particularly in Africa [3,4]. In a recent study conducted in rural Burkina Faso, a West African nation, 21% of adolescents and 61% of adults exhibited some degree of periodontitis. Limited access to health services due to financial constraints and a lack of education appears to be a significant contributing factor. Remarkably, 78% of adults in this study had never visited a dentist [5]. Consequently, improving access to oral health prevention and care for these populations represents a critical public health issue.

Periodontitis is initiated by the accumulation of bacterial biofilm on tooth surfaces, which triggers and maintains an exaggerated inflammatory response. Consequently, topical antimicrobial and anti-inflammatory drugs have been proposed as adjuncts to mechanical periodontal treatment, encompassing oral hygiene improvement and professional instrumentation. Bioactive natural compounds in herbal medicines can provide safe, inexpensive, and effective therapeutic alternatives to conventional drugs with minimal side effects. It is worth noting that approximately 80% of the world’s population currently uses herbal medicines to meet their primary healthcare needs, particularly in rural areas of developing countries [6].

The growing interest in herbal medicines has resulted in rapid inflation in research in this field in recent years. Thus, the number of randomized controlled trials on herbal/natural products increased by almost 150% between 2013 and 2015 only [7]. The most studied oral conditions included periodontal diseases such as gingivitis and periodontitis [7]. Recently, a large panel of herbal medicines studied over the last five years in randomized clinical studies was comprehensively reviewed [8]. Most of these natural products, such as *Aloe vera*, *Curcuma longa*, *Punica granatum*, and *Salvadora persica*, have demonstrated antimicrobial and anti-inflammatory properties, which may explain their benefits as topical products such as toothpaste gels and mouthwashes in the treatment of gingivitis and periodontitis [8]. For example, curcumin, the main compound in turmeric (*Curcuma longa*), shows antibacterial activity against oral bacteria, including periodontopathogens, as well as promising anti-inflammatory, immunomodulatory, antioxidant, and wound-healing properties for periodontal application [9]. Turmeric mouthwashes or gels have provided similar outcomes to chlorhexidine in terms of plaque and gingival inflammation reduction in several clinical studies [10-12].

Among the traditional remedies documented in Burkina Faso, previous ethnobotanical surveys have identified three plants, namely *Cochlospermum tinctorium* (*C. tinctorium*), *Flueggea virosa* (*F. virosa*), and *Waltheria indica* (*W. indica*), frequently recommended by traditional practitioners for addressing “loosening of teeth” or “gum bleeding” [13].

*C. tinctorium* is a perennial savannah herb characterized by a robust, fibrous stump. Annually, it produces unbranched cylindrical aerial stems measuring 50 to 100 cm in height, featuring bark that can be peeled off in fibrous strips and a distinctive aroma. The leaves, alternate and petiolate, exhibit deep palmate morphology with five narrowly lanceolate, finely denticulate lobes. The large, yellow, actinomorphic flowers terminate in pointed tips and measure 10 to 12 cm in width. The dehiscent, capsular fruits are dehiscent; the capsular are ovoid and reach lengths of up to 6 cm, opening through four valves. The seeds are densely covered with long hairs. This plant acts as a pioneer species that regenerates after bushfires [14].

*F. virosa* is a shrub reaching up to six meters in height with a smooth gray-brown bark, occasionally marked by cracking or roughness. Its leaves are alternate and simple, measuring 1.5 to 2 mm in length and displaying nearly orbicular to obovate or elliptical shapes, with a wedge-shaped to rounded base and an obtuse, rounded, or emarginate apex. The flowers, unisexual and regular, are comprised of five merged parts and emit a fragrance. The fruits, slightly fleshy, manifest as three-lobed, globular capsules, measuring 3 to 5 mm in diameter, which subsequently dehisce, revealing up to six shiny, oval yellowish-brown seeds, each spanning 2 to 3 mm in length [15].

*W. indica*, on the other hand, is a short-lived shrub that grows to heights of one to two meters. It features a cylindrical, solid stem that is woody at the base. The root is flexible and can be uprooted. The leaves, simple and alternate, are dense, slightly pubescent, and serrated. The flowers are organized in constricted cymes resembling glomeruli, which are either axillary or situated at the branch ends. The plant begins to flower at approximately six months of age and continues to flower more or less continuously until its demise. The fruits have dehiscent capsules containing a single seed [16].

In the context of this scoping review, we aimed to summarize current knowledge on the potential antimicrobial and anti-inflammatory properties of these three plants, as well as to elucidate their bioactive components. This endeavor seeks to establish a foundational basis for the scientific rationale behind their therapeutic application in the management of periodontitis.

## Review

Materials and methods

This review adheres to the PRISMA guidelines extension for scoping reviews (PRISMA-ScR) [17]. A systematic electronic search encompassing three databases, namely PubMed, Science Direct, and Google Scholar, was conducted up until July 2022. The search query employed was as follows: (*Waltheria indica*) OR (*Fluggea virosa*) OR (*Cochlospermum tinctorium*) OR (*Securinega virosa*) OR (*Flueggea virosa*). Additionally, a manual search was undertaken using the bibliographic references of the selected articles. The inclusion criteria encompassed preclinical, in vitro, in vivo, and experimental clinical studies investigating the antimicrobial and/or anti-inflammatory effects of the selected plants. Conversely, literature reviews and ethnobotanical studies were excluded. The article selection process was executed independently by two reviewers (AD and KA), with any discrepancies being resolved through discussion. The extracted information from the included studies encompassed the first author, publication year, type of study, part of the plant used, extraction method, identified active compound(s), target molecule(s) or pathogen(s), and main results. Due to the expected heterogeneity of the data and the paucity of experimental studies in humans, only a qualitative synthesis of the data could be provided.

Results and discussion

**Figure 1:**
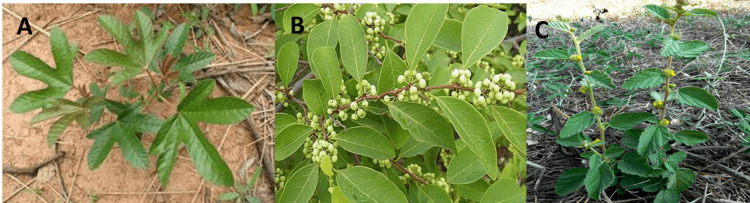
Plant photography A: *C. tinctorium*; B: *F. virosa*; C: *W. indica* Image Credit: Authors

Figure 1 represents a photograph of the three plants in their natural habitat.

**Figure 2:**
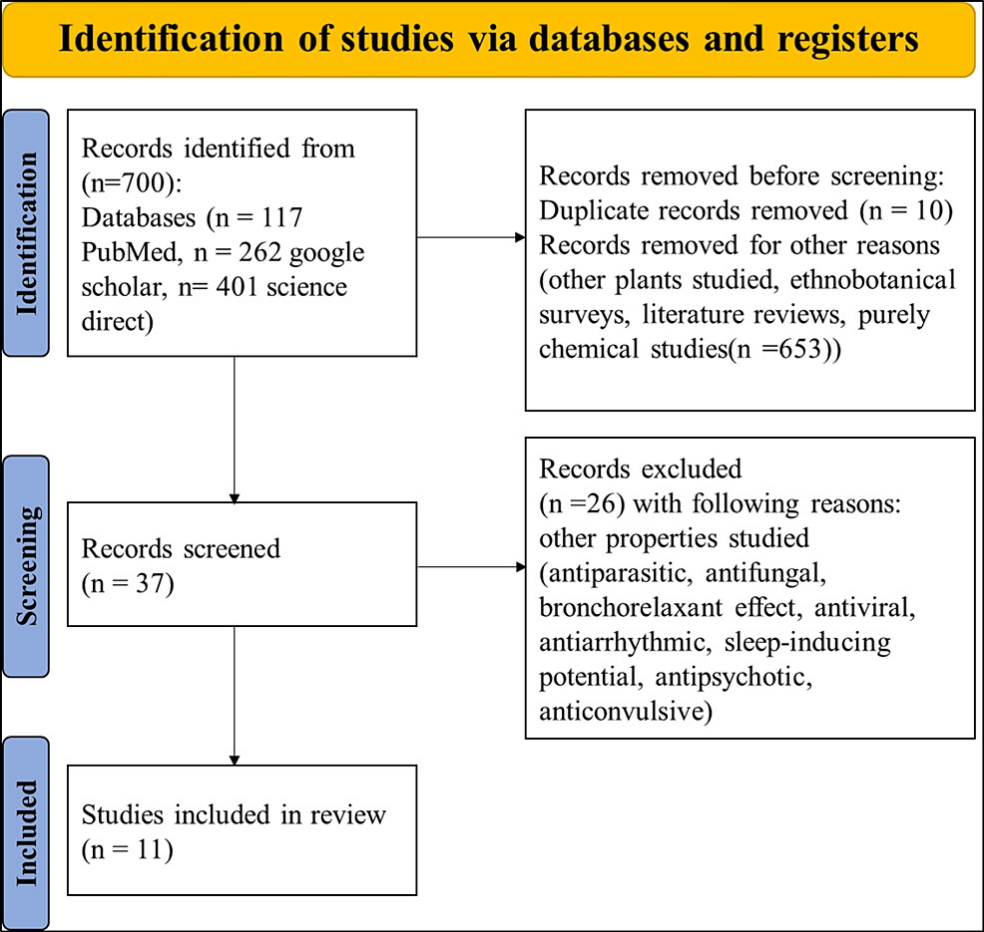
Flowchart of the study selection process

From the initial pool of 700 articles, a total of 11 studies were ultimately incorporated (Figure 2) [13,18-27]; the majority of these (80%) of these were in vitro studies.

**Figure 3:**
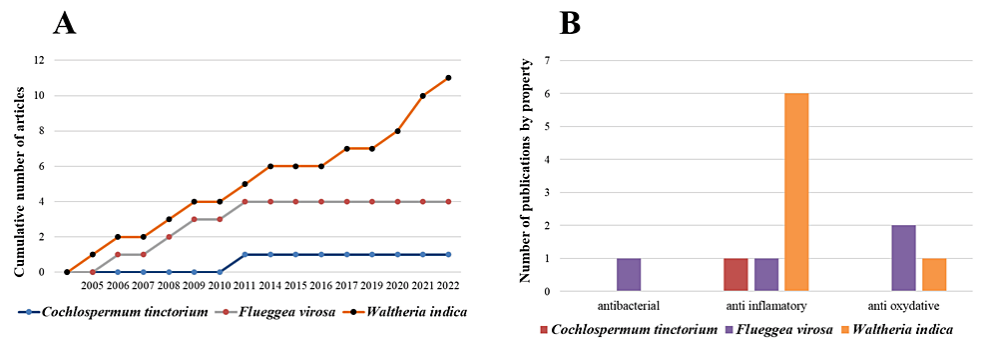
Timeline of the publications and main outcome according to the type of plant A: Indexed publications on the pharmacological properties of *C. tinctorium*,*F. virosa*,* and W indica*. B: Distribution of articles according to the studied properties of *C. tinctorium*,* F. virosa*,* and W. indica* Image Source: Authors

Figure 3 shows the evolution over time of the number of publications pertaining to the pharmacological properties of the three plants over a period up to June 2022.

Among the trio of plants, *W. indica* emerged as the most extensively scrutinized, accounting for 65% of the studies. The plant parts used predominantly comprised aerial sections (leaves) or roots. In the case of *C. tinctorium*, extracts were derived from leaves, roots, and rhizomes, whereas for *F. virosa*, extracts from leaves, root barks, and rhizomes were used. In the case of *W. indica*, two studies used leaf extracts, two used aerial extracts, three used root extracts, and one used whole plant extracts. Several chemical compounds have been characterized and isolated from our plants of interest. Thus, *C. tinctorium* houses a plethora of compounds such as carotenoids, tannins (such as gallic acid, ellagic acid, and ellagitanin), and essential oils (e.g., 3-hexadecanone) [28]. Moreover, it harbors molecules such as triacylbenzenes (cochlospermines A, B, C, D, and 1,3-bis(tetradecanoy1), alphitolic acid, 1-hydroxytetradecan-3-one, and β-bisabolene [29,30]. Phytochemical analyses of *F. virosa* have revealed the presence of alkaloids, flavonoids, sterols, terpenes, saponins, tannins, and carbohydrates [31]. Meanwhile, the analysis of the major chemical compounds of *W. indica* has highlighted the predominance of alkaloids, flavonoids, saponosides and tannins, along with a roster of molecules like epicatechin, waltherione L, waltherione F, betulinic acid, and quercetin [32]. These compounds have potential pharmacological properties that could be used in the treatment of various diseases (Table 1).

**Table 1:** Main characteristics of the studies included in the scoping review NR: not reported

Primary author, year of publication	Type of study	Plant (part used)	Extraction method	Identified active compound(s)	Target molecule(s) or pathogen(s)	Main results
Liu et al., 2022 [18]	Analytical/ in vitro	*Waltheria indica* L. (roots)	Methanolic extract	Seven new coumarinolignans, walthindicins A–F (1a, 1b, 2–5, 7), five known analogs (6, 8–11)	ROS/NF-κB	Compounds 1a and 6 showed superior ROS inhibitory activity at 20 μg/mL in HeLa cells. Compounds 1a and 6 showed moderate NF-κB inhibitory activity in a concentration-dependent manner in Luc-HEK-293 cells
Termer et al., 2021 [19]	In vitro (analytical)	*Waltheria indica *L. (leaves)	Accelerated solvent extraction (Dionex ASE) 350/ethanol extracts	Alpha-linolenic acid; linoleic acid; oleic acid	PGE2 by inhibiting COX-2	Fatty acids are responsible for up to 41% of the COX-2 inhibition. Compounds contribute to COX-2 inhibition in the order linoleic acid > alpha-linolenic acid > steroidal saponins > triterpenoid saponins
Termer et al., 2021 [20]	In vitro (analytical)	*Waltheria indica *L. (leaves)	Accelerated solvent extraction (Dionex ASE) 350/ethanol extracts	Tiliroside (flavonoid)	COX-2 (inhibition)	Waltheria Indica extracts inhibit the inflammatory key mediator COX-2. The activity is related to the extraction parameters governing the composition of the extract
Laczko et al., 2020 [21]	In vitro	*Waltheria indica *L. (aerial parts)	80% ethanolic extract	NR	IL‑1B, TNF‑α, TNFRII and NF‑κB	Waltheria extracts inhibit the expression of key inflammatory cytokines and cytokine receptors including IL-1B, IL-1ra, IL-8, and IL-6, and additionally, through reduced expression of TNF-α and its receptor TNF RII, inhibit TNF-α- associated pro-inflammatory signaling
Monteillier et al., 2017 [22]	In vitro	*Waltheria indica *L. (root and aerial parts)	Dichloromethane decoction extract	Alkaloids/triterpenes/flavonoids	NF-κB/ QR (quinone reductase)	*W. indica* contains both NF-κB inhibitors and QR-inducing compounds. The decoction and the alkaloid extract were active at 20 μg/ml with 51% and 79% inhibition (NF-κB)
Zongo et al., 2014 [13]	In vitro/in vivo	*Waltheria indica* L. (roots)	Hydroalcoholic extract	Epicatechin isomer	PDE4A1α, PLA2, 5-LOX (inhibition)	Extracts and (-)-epicatechin isolated from roots of *W. indica* reduced PDE4A1α, 5-LOX, and PLA2 activities
Ahmed et al., 2011 [23]	In vitro/in vivo	*Cochlospermum tinctorium.* (leaves, roots, and root barks)	70% aqueous methanolic extracts	Alkaloids, saponins, tannins, flavonoids	NR	The aqueous methanol root, leaf, and root bark extracts of Cochlospermum tinctorium possess analgesic and anti-inflammatory activities in laboratory animals. Flavonoids, saponins, and tannins may be responsible for these effects
Sanogo et al., 2009 [24]	In vitro analytical	*Flueggea virosa* (Willd.) Voigt Syn: *Securinega virosa*, Roxb. and Willd. (leaves)	Methanolic extract	3-O-kaempferol 4-O-(galloyl)-β-D-glucoside/11-0caffeoylbergenin	NR	Kaempferol 3-O-(4-galloyl)-β-D-glucopyranoside (1), 11-0-caffeoylbergenin (2), and glucogallin (6) exhibited the highest antioxidant capacity, being also able to modulate hydroxyl radical formation more efficiently than the other compounds, acting as direct hydroxyl radical scavengers and chelating iron
Magagi et al., 2008 [25]	In vivo	*Flueggea virosa* (Willd.) Voigt Syn: *Securinega virosa*, Roxb. and Willd. (root barks)	Methanolic extract	Flavonoids, saponins, tannins, glycosides, alkaloids and steroids	NR	The methanolic root bark extract of SV (6.25-25 mg kg-1 body weight, i.p.) significantly (P<0.05) inhibited acetic acid-induced abdominal constrictions and attenuated the neurogenic pain (phase 2) induced by formalin. The extract also significantly (P<0.01) prolonged the reaction latency to pain thermally induced in mice by the hot plate. The extract at the doses (6.25, 12.5, and 25 mg kg-1) tested afforded 12%, 52%, and 52% inhibition of paw edema, respectively, at the end of the third hour. The intraperitoneal and oral LD50 values in mice were found to be 774.6 and greater than 5000 mg kg-1, respectively
Dickson et al., 2006 [26]	In vitro	*Flueggea virosa* (Willd.) Voigt Syn: *Securinega virosa*, Roxb. and Willd. (root barks)	Petroleum spirit, chloroform, and ethanol extracts	NR	*Micrococcus flavus*, *Bacillus subtilis*, *Staphylococcus aureus*, multidrug-resistant *S. aureus*, tetracycline-resistant *S. aureus*, erythromycin-resistant *S. aureus*, *Streptococcus faecalis*, *Salmonella*, *Pseudomonas aeruginosa*, *Escherichia coli*, Klebsiella aerogenes, *Candida albicans*, *Saccharomyces cerevisiae*, *Trichophyton interdigitale*, and *Microsporum gypseum*	The chloroform extract of *S. virosa* was the most active extract showing activity against 13 test organisms with MIC values ranging from 15.6 μg/mL to over 1000 μg/mL. The petroleum spirit, chloroform, and ethanol extracts of *S. virosa* exhibited modulation effects when combined with standard antibiotics against resistant strains of *S. aureus*
Rao et al., 2005 [27]	In vitro	*Waltheria indica* L. (whole plants)	80% aqueous-ethanolic extract	Flavonoids (( )-epicatechin, quercetin, and tiliroside)	NO, TNF-alpha, and IL-12	Flavonoid compounds significantly and dose-dependently inhibited the production of the inflammatory mediators NO, TNF-α, and IL-12 in activated macrophages

This scoping review highlighted the use of *C. tinctorium, F. virosa, and W. indica* for their anti-inflammatory and antibacterial properties. According to our search criteria, *W. indica* has generated the highest number of publications (seven during the review period), reflecting the pronounced scientific interest in this plant. In contrast, publications concerning the other two plants have followed a linear trajectory over the last decade and remain limited in number. *W. indica* has been studied more extensively for its anti-inflammatory properties, with only one focusing on its antibacterial properties, namely the research by Dickson et al. in 2006, which evaluated the antibacterial potential of *F. virosa* [26].

Additionally, two other studies evaluated the antioxidant properties of* F. virosa* [24] and *W. indica* [18]. In contrast, very little information is available regarding the pharmacological properties of *C. tinctorium*. In fact, of the 11 articles included in our study, only one dealt with this plant, examining its anti-inflammatory potential and other properties that fall outside the subject of our investigation [23].

Pharmacological properties

Anti-inflammatory properties of C. tinctorium, F. virosa, and W. indica

Inflammation is a key factor in periodontal disease [33]. Indeed, chronic inflammation, as observed in periodontitis, culminates in the sequential synthesis of pro-inflammatory mediators, including prostaglandins, leukotrienes, and cytokines (e.g., tumor necrosis factor α (TNF-α), interleukin 1β (IL-1β), interleukin 6 (IL-6), interferon γ (INFγ), and prostaglandin E (PGE)) [34].

It has been reported in the literature that the extracts of our focal plants are rich in polyphenols, particularly flavonoids, which possess the ability to inhibit pro-inflammatory enzymes [35,36]. The anti-inflammatory activity of flavonoids was first described in 1980 by Baumann et al. [37]. These compounds are able to regulate the enzymatic activity of arachidonic acid (AA), a fatty acid released during inflammation, through the inhibition of phospholipase A2 (PLA2), cyclooxygenase (COX), lipoxygenase (LOX), and prostaglandin endoperoxide hydrogen synthase (PGHS) [20,38,39]. The inhibition of LOX and COX by these extracts could contribute to reducing the increased release of pro-inflammatory cytokines (Il-1β, Il-6, IL-10, TNFα, INFγ, PGE2, etc.) as well as metalloproteinases (MMPs) [40,41].

In our investigation, only one study addressed the anti-inflammatory effects of *C. tinctorium*, conducted by Ahmed et al. in 2011 [23]. In this study, the authors demonstrated the ability of *C. tinctorium* to significantly reduce paw edema in rats during the carrageenan edema test. Phytochemical screening of *C. tinctorium* revealed the presence of alkaloids, flavonoids, tannins, and cardiac glycosides [42]. Flavonoids, tannins, and saponosides have been reported to possess antibacterial, anti-inflammatory, and analgesic properties [43]. These compounds are seemingly present in the aqueous and methanolic extracts of *C. tinctorium*, which may substantiate its empirical use in traditional medicine.

In the case of *W. indica*, it contains several active compounds. Quantification of these chemical groups in *W. indica* leaves underscores the presence of alkaloids, flavonoids, saponosides, and tannins [44]. These molecules contribute to the plant’s diverse pharmacological properties. In fact, Rao et al. demonstrated that *W. indica* leaves exhibit analgesic and anti-inflammatory attributes owing to the presence of polyphenolic compounds, especially flavonoids [27].

There has been little discussion about *F.virosa*'s anti-inflammatory properties. Only Magaji et al. in 2008 indicated that methanolic extracts of *F. virosa* roots significantly reduced rat paw diameter in the carrageenan-induced paw edema test. A preliminary phytochemical screening conducted in this study unveiled the presence of alkaloids, tannins, saponins, and flavonoids in these roots [25].

Antibacterial Properties of C. tinctorium, F. virosa, and W. indica

Experimental research supports the antimicrobial effect of our focal plants against a variety of pathogens, encompassing bacteria, viruses, parasites, and fungi, among others [26,45-47]. Although we found no direct evidence of this effect against periodontal pathogens, the literature attests to the antibacterial effect of several polyphenol-rich plants against periodontal bacteria [48]. Indeed, Sanchez et al. in 2019 demonstrated that applying polyphenols derived from red wine to a 72-hour periodontal biofilm for one to five minutes resulted in a significant reduction in the viability of periodontal bacteria, including *Aggregatibacter actinomycetemcomitans *(*A. actinomycetemcomitans*)* and Porphyromonas gingivalis *(*P. gingivalis*)* *[49]. Notably, periodontal disease's primary etiological factor is a biofilm, a complex bacterial consortium with the most pathogenic species clustered within the Sockransky red complex and *A. actinomycetemcomitans* [50]. In general, plants contain phytochemicals such as alkaloids, essential oils, flavonoids, and tannins, which exhibit potent antimicrobial activity and are used as anti-inflammatory, antibiotic, analgesic, and antioxidant agents [51].

Polyphenols are endowed with great antimicrobial and immunomodulatory potential in the treatment and prevention of periodontal disease [52]. Our focal plants, in particular, *C. tinctorium*,* F. virosa*,* and W. indica,* contain tannins and flavonoids, both of which belong to this chemical group. Tannins participate in numerous biological processes within the body, including antimicrobial activity, which appears to be attributed to their capacity to inactivate microbial adhesins, enzymes, cell envelope transport proteins, and more. Flavonoids, on the other hand, appear to be active against several viruses and many periodontal bacteria, including *P. gingivalis*, *A. actinomycetemcomitans*, *Fusobacterium nucleatum*, and *Prevotella intermedia* [52,53].

Furthermore, the antibacterial activities of securin and viroallosecurnin, alkaloids of *S. virosa* leaves have also been reported previously. In our investigation, chloroform and ethanolic extracts of *F. Virosa* yielded favorable outcomes against several bacteria, including *Staphylococcus aureus* [26]. These findings bolster the rationale for experimental studies using our plant extracts against periodontal biofilm models.

Antioxidant Activity of C. tinctorium, F. virosa, and W. indica

The three plants under scrutiny possess antioxidant properties attributable to their high polyphenolic compound content. For instance, Garba et al. in 2012 reported that hexane extracts from *W. indica* leaves (1.0 mg/mL) exhibited higher antioxidant activity (92.8%) than ascorbic acid (90.2%) and α-tocopherol (15.4%) [54]. This activity was attributed to flavonoids and tannins, which function as scavengers of free radicals.

Among the mechanisms implicated in the pathophysiology of periodontitis, the up-regulation of reactive oxygen species could play a pivotal role in the initiation and progression of periodontitis and other inflammatory diseases through the development of oxidative stress [55]. Periodontal bacterial aggression leads to the predominant recruitment of polymorphonuclear PMNs, which form the first line of cellular defense against periodontal pathogens [56]. Notably, a specific phenotype of polymorphonuclear neutrophils known as "hyperactivated" appears to be associated with periodontal disease. This phenotype is characterized by an overproduction of reactive oxygen species and proteases, rendering patients with elevated levels of this phenotype more likely to develop periodontitis [57,58]. The antioxidant activity exhibited by our focal plants holds therapeutic potential in preventing the oxidative stress involved in periodontitis.

Toxicological Data on C. tinctorium, F. virosa, and W. indica

Several studies have underscored the favorable tissue tolerance of the three plants. Diallo et al. in 1992 demonstrated that aqueous, hydroethanolic, and ethanolic extracts of *C. tinctorium* rhizome had hepatoprotective activity in vitro and in vivo in mice. These extracts significantly inhibited lipid peroxidation and hepatocyte lysis induced by tert-butyl hydroperoxide and carbon tetrachloride, respectively [28]. Notably, the oral lethal dose (LD50) of *C. tinctorium* administered orally has been estimated to exceed 3000 mg/kg in rats, with demonstrated cytoprotective effects on liver cells and the K562 cell line [59,60].

Aqueous extract of *F. virosa* yielded an oral LD50 ranging from 200 to 500 mg/kg in rats [61]. Moreover, Adedapo et al. in 2007 found that the median LD50 by the oral route of *F. virosa* extract exceeded 3000 mg/kg. Treatment with this extract exerted no discernible effect on the central or autonomic nervous systems. Additionally, the hydroalcoholic extract of *F. virosa* stem bark did not cause significant changes in the macro-anatomical structure of the liver, kidneys, and lungs. Furthermore, the relative weights of the liver, kidneys, spleen, and thymus remained unaltered in response to the extract [62].

Aqueous extracts derived from the roots, stems, and leaves of *W. indica* yielded intraperitoneal lethal doses (LD50) of 69 mg/kg, 141 mg/kg, and 363 mg/kg body weight in mice, respectively [63]. Hamidu et al. in 2008 found that the hydroethanol extract of the plant's aerial parts exhibited mild toxicity, with an intraperitoneal LD50 of 875 mg/kg body weight in mice [64]. Additionally, the aqueous extract of *W. indica* leaves elicited an oral LD50 exceeding 2000 mg/kg in rats [16]. In conclusion, these findings indicate that the three plants exhibit no toxicity at typical therapeutic doses when administered orally; however, high doses should be used with caution, especially in the case of *W. indica*.

## Conclusions

This review highlighted the antimicrobial potential of *C. tinctorium*, *F. virosa*, and *W. indica* as well as their beneficial effects on modulating a number of inflammatory pathways involved in periodontitis. Although none of the studies leveraged validated models of periodontitis or examined these plants against periopathogens, their dual capacity for anti-inflammatory and antibacterial actions, in conjunction with their various biological activities, augments their promise for employment in the treatment of periodontal disease. Experimental studies in vitro and on animal models are needed to confirm the potential of these plants and justify their use in traditional medicine.
